# A standardized universal protocol for using adjunct abdominal ultrasound at the time of diagnosis for suspected necrotizing enterocolitis

**DOI:** 10.1007/s00247-025-06408-x

**Published:** 2025-10-10

**Authors:** Clifford Hegedus, Clare Essex, Katherine Vincent, Alanna Shiflett, Leslie Spence, Jeanne Hill, Katherine E. Chetta

**Affiliations:** 1 C.P. Darby Department of Pediatrics, Division of Neonatal-Perinatal Medicine, Medical University of South Carolina, 10 McClennan Banks Drive, MSC 915 SJCH Room 2190, Charleston, SC 29425 United States; 2 Medical University of South Carolina, Charleston, United States; 3 Department of Pediatric Radiology, Shawn Jenkins Children’s Hospital, Medical University of South Carolina, Charleston, United States; 4 C.P. Darby Children’s Research Institute, Shawn Jenkins Children’s Hospital, Medical University of South Carolina, Charleston, United States

**Keywords:** Enterocolitis, necrotizing, Infant, newborn, Infant, premature, Intensive care units, neonatal, Radiography, abdominal, Ultrasonography

## Abstract

**Background:**

Necrotizing enterocolitis (NEC) remains a highly morbid disease for preterm infants. The use of adjunct abdominal ultrasonography improves the evaluation of early or uncertain cases of NEC. No institutions have reported implementing a standardized adjunct abdominal ultrasound guideline and universally adopting adjunct abdominal ultrasound in routine neonatal practice for the evaluation of NEC.

**Objective:**

To determine if a standardized, adjunct abdominal ultrasound guideline for diagnosing NEC is feasible and beneficial for the routine detection of pathologic abdominal findings in NEC.

**Materials and methods:**

This retrospective study was conducted in an 82-bed level IV academic neonatal intensive care unit from February 2023-April 2024. Unit guidelines were updated in February 2023 to recommend the addition of an adjunct abdominal ultrasound to evaluate NEC universally at the time of initial evaluation. Imaging data was abstracted from a post-implementation observation period of 15 months. Implementation feasibility was assessed. Sonographic findings consistent with NEC were described after implementing standardized sonography protocols.

**Results:**

Adjunct abdominal ultrasound was performed in 22 of 23 cases (96%) of NEC following guideline implementation. Twenty ultrasounds (90%) had at least one finding suggestive of NEC, and 12 (55%) had multiple findings suggestive of NEC. Seven ultrasounds (32%) showed findings of NEC in cases with unremarkable initial abdominal radiographs. Two NEC cases had radiographic findings without abdominal ultrasound findings. The sensitivity of abdominal ultrasound for NEC was 0.91. In 59% of cases, abdominal ultrasound findings resulted in a higher modified Bell’s stage when compared to initial abdominal radiograph findings.

**Conclusion:**

Implementing a standardized adjunct abdominal ultrasound guideline to assist with the initial diagnostic evaluation of NEC is feasible and may aid in diagnosis of NEC.

**Graphical abstract:**

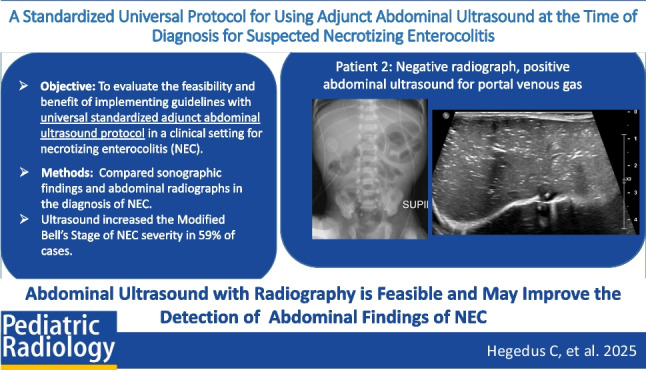

**Supplementary Information:**

The online version contains supplementary material available at 10.1007/s00247-025-06408-x.

## Introduction

Necrotizing enterocolitis (NEC) is a severe gastrointestinal condition of preterm infants and results in significant morbidity and mortality. Low birth weight preterm infants and full-term infants with cyanotic heart disease are also at an elevated risk for NEC [1–4]. The exact causes of NEC remain unclear, although the pathogenesis includes excessive inflammation, impaired intestinal barrier function, intestinal dysbiosis, hypoxia, and bacterial translocation, resulting in sepsis [4–8]. NEC is treated with bowel rest (NPO or “nil per os”), parenteral nutrition, and antibiotics (“medical NEC”), or surgical management with peritoneal drain placement or laparotomy with possible bowel resection (“surgical NEC”). Overall mortality for infants with confirmed NEC is between 15–30%, while mortality for infants with surgical NEC may be as high as 50% [9–13].

Due to the high morbidity and mortality of NEC, the early diagnosis, staging, and treatment of NEC remain urgent areas of research. A study by Chetta et al. showed that delays in antibiotic treatment might be associated with more severe NEC outcomes [14]. Abdominal radiography is considered the gold standard imaging modality for diagnosing NEC. However, some evidence suggests that abdominal ultrasonography may be a more sensitive tool for specific intra-abdominal findings of NEC.

Abdominal ultrasonography may be a more sensitive modality than plain radiography for detecting pneumatosis intestinalis and portal venous gas, which are crucial for both diagnosis and staging of NEC [3, 5, 15–17]. Sonography exceeds the diagnostic capabilities of abdominal radiography in several areas. Unlike abdominal radiographs, abdominal ultrasonography can detect complex ascites and decreased or absent peristalsis, characterize changes in bowel wall thickness, and evaluate intestinal perfusion [15]. Other studies have suggested that abdominal ultrasonography may predict the need for surgical intervention or an increased risk of mortality [2, 3, 18–20]. Currently, many institutions reserve the use of abdominal ultrasonography for evaluating NEC when abdominal radiograph findings or clinical findings are equivocal. To the best of our knowledge, no institutions in the United States have reported implementing a standardized adjunct abdominal ultrasound guideline and universally adopting adjunct abdominal ultrasound in routine neonatal practice for the evaluation of NEC.

The primary aim of this study was to evaluate the feasibility and benefit of implementing guidelines for the universal screening of NEC with a standardized adjunct abdominal ultrasound protocol in a neonatal intensive care setting.

## Materials and methods

This retrospective study included all infants with confirmed necrotizing enterocolitis at our Level IV academic neonatal intensive care unit (NICU) between February 2023 (guideline implementation) and April 2024. The Institutional Review Board approved this study, and consent was waived.

**Fig. 1:**
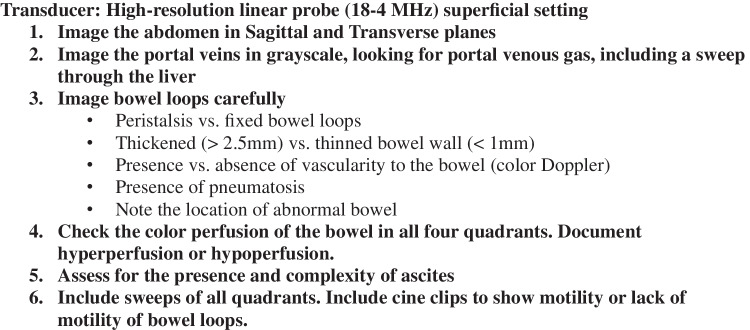
Limited abdominal ultrasound for necrotizing enterocolitis sonographer protocol

A work group consisting of a Neonatologist, Pediatric Radiologist, and Neonatal-Perinatal Medicine fellow fully standardized the approach to using abdominal ultrasound to diagnose NEC. The unit guideline for the management of NEC includes an evaluation and treatment plan consisting of an array of laboratory tests, blood cultures, antibiotics, serial radiographs, and empiric antibiotic management based on modified Bell’s staging criteria (Online Resource 1). Providers are instructed that in cases of NEC with high clinical suspicion, the unit guideline is to be rapidly and fully implemented, including all diagnostic tests and initiation of antibiotics. Before this study, our unit’s guidelines for evaluating NEC relied solely on abdominal radiography for diagnostic imaging. In February 2023, these guidelines were updated to recommend that clinicians obtain a limited abdominal ultrasound with the indication: “for the evaluation of NEC” during the initial workup of NEC, in addition to radiography. All technologists performing the exams were certified pediatric sonographers and were available to perform scans 7 days a week, 24 h a day. Attending pediatric radiologists reviewed images and were available 24 h a day. Two ultrasound machines were used during this time: Epiq Elite and Epiq 5G (Philips, Bothwell, Washington). The sonographer’s protocol for the indication “evaluation of NEC” was standardized (Fig. 1).

A dictation template corresponding to the protocol outline was created (Online Resource 2). The dictation template included specific sections for commenting on the presence or absence of seven findings indicative of NEC: pneumatosis intestinalis, portal venous gas, pneumoperitoneum, bowel wall thickening or thinning, intra-abdominal complex fluid, decreased peristalsis, and changes in bowel perfusion.

**Fig. 2:**
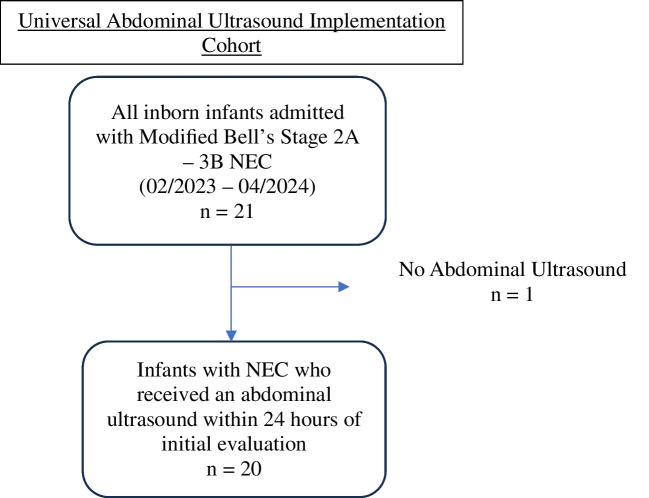
Flowchart showing patient selection

An independent chart review verified each case of NEC before it was included in the analysis. Patients were excluded if they were transferred to our center for NEC or if the initial evaluation for NEC had begun at an outside center prior to transport. Infants with a diagnosis of modified Bell’s stage 1 NEC were also excluded; all cases included in analyses received modified Bell’s staging of 2A-3B (Fig. 2) [21].

Demographic information was collected, including birth weight, gestational age, sex, modified Bell’s stage, and whether the case of NEC was medical, surgical, or resulted in death. The length of each abdominal ultrasound study was also collected. Infants were excluded from the surgical NEC outcome group if a laparotomy was performed non-emergently after the acute episode of NEC, such as for the treatment of bowel stricture.

The demographic data, as well as the frequency of NEC findings on abdominal ultrasounds, were described for all infants with modified Bell’s stage 2A–3B NEC between February 2023 and April 2024, who universally received an adjunct abdominal ultrasound with their initial diagnostic evaluation. The sensitivity of abdominal radiography and adjunct abdominal ultrasound for the diagnosis of NEC was calculated. The specificity of abdominal radiography and adjunct abdominal ultrasonography was not calculated as this study did not capture negative cases of NEC.

## Results

There were 23 total cases of NEC with modified Bell’s stage 2A–3B following guideline implementation over a 15-month period. Two of these cases of NEC occurred in patients with a prior history of NEC. Three (15%) cases resulted in an outcome of surgery and/or death. Only one case of NEC did not have an abdominal ultrasound obtained during the initial diagnostic evaluation. Compliance with the guideline was 96%. Twenty-two episodes of NEC in twenty patients, including the two repeat episodes of NEC, received adjunct abdominal ultrasounds during the first assessment, following the implementation of institutional guidelines, and were included in the analysis. The single case of NEC that did not have an ultrasound performed required urgent procedural intervention due to rapid clinical deterioration. The average length of the abdominal ultrasounds was 13±8 min. Five (23%) ultrasounds were performed between the hours of 19:00 and 07:00. No complications or difficulties were encountered by the pediatric sonographers, Pediatric Radiologists, or NICU providers regarding the implementation of the protocol. There were no reported errors in obtaining the ultrasound images.

Of the 20 infants with NEC who received an abdominal ultrasound, the average birth weight was 1,016±467 g, and the average gestational age was 28±4 weeks (Table 1).

**Table 1:** Baseline characteristics

Baseline characteristics		Abdominal ultrasound cohort *N*=20 (%)
**Birth weight (grams)**		1,016±467
**Gestational age (weeks)**		28±4
**Female sex**		7 (35%)
NEC modified Bell’s stage*	**2A**	7 (35%)
	**2B**	5 (25%)
	**3A**	5 (25%)
	**3B**	3 (15%)

*Stage reported as highest grade if multiple episodes in a single patient

Table 2 describes the abdominal ultrasound findings in cases of NEC following protocol implementation.

**Table 2:** Frequency of abdominal ultrasound findings per episode of necrotizing enterocolitis

Sonographic finding	Cases of NEC with abdominal ultrasounds ***n***=22* (%)
**Pneumatosis intestinalis**	13 (59%)
**Portal venous gas**	10 (45%)
**Pneumoperitoneum**	1 (5%)
**Bowel wall thickening or thinning**	6 (27%)
**Complex free fluid**	5 (23%)
**Decreased peristalsis**	4 (18%)
**Bowel perfusion change**	4 (18%)
**Any findings**	20 (91%)
**Multiple findings**	12 (55%)

*Two patients with repeat cases of NEC

Twenty out of twenty-two cases of NEC (91%) had at least one ultrasound finding of NEC, and 12 cases (55%) had multiple ultrasound findings consistent with NEC. Of all cases with abnormal abdominal ultrasound findings, 7 (32%) cases had initial abdominal radiographs without findings of NEC (Table 3). The sensitivity of ultrasound in NEC was 0.91. The sensitivity of the initial abdominal radiography for NEC was 0.68.

**Table 3:** Agreement for abdominal ultrasound and radiography for necrotizing enterocolitis

NEC cases (***n***=22)		Radiographic findings	
		Present	Absent
**Ultrasound findings**	**Present**	13* (59%)	7 (32%)
	**Absent**	2 (9%)	0

*Includes 5 cases (23%) with indeterminate or possible pneumatosis intestinalis as the only radiographic finding

These 7 NEC cases without abdominal radiographic findings do not include the 6 (27%) additional radiographs with the only reported finding of lucencies indicative of possible or indeterminate pneumatosis intestinalis. In 13 (59%) cases, abdominal ultrasound findings resulted in a higher modified Bell’s stage when compared to initial abdominal radiograph findings. Table 4 describes the radiograph and ultrasound findings for each of these 13 cases, as well as the change in modified Bell’s stage.

**Table 4:** Imaging findings of necrotizing enterocolitis cases with upgraded modified Bell’s stage

Case	Abdominal radiograph findings (time)	Abdominal ultrasound findings (time)	Time between radiograph and ultrasound (minutes)	Upgraded (initial* and final) modified Bell’s stage
1	Pneumatosis (22:01)	Pneumatosis, pneumoperitoneum, complex free fluid (00:59, after midnight)	178	2 A to 3B
2	No findings (21:33)	Portal venous gas (22:04)	31	None to 2B
3	Pneumatosis, portal venous gas (08:06)	Pneumatosis, portal venous gas, bowel thickening, decreased perfusion, free fluid collection (09:33)	87	2B to 3A
4	Indeterminate pneumatosis/lucencies (12:59)	Portal venous gas (05:32, after midnight)	633	1B to 2B
5	No findings (14:23)	Pneumatosis (16:44)	141	None to 2A
6	No findings (08:06)	Pneumatosis, portal venous gas (11:07)	181	None to 3 A for severe metabolic and lactic acidosis
7	No findings (16:25)	Pneumatosis, bowel thickening, increased perfusion _(12:11)	254	None to 2A
8	Pneumatosis, portal venous gas (14:11)	Pneumatosis, portal venous gas, absent peristalsis, decreased perfusion, complex free fluid (12:07)	124	2B to 3 A for complex free fluid with severe metabolic and lactic acidosis with hypotension
9	Indeterminate pneumatosis/lucencies (00:41)	Pneumatosis, bowel thickening (01:56)	75	1B to 2A
10	Pneumatosis (06:53)	Pneumatosis, portal venous gas, bowel thickening, complex free fluid suggestive of bowel perforation (08:18)	85	2 A to 3B
11	No findings (11:35)	Portal venous gas (12:22)	47	None to 2B
12	Indeterminate pneumatosis/lucencies (19:55)	Pneumatosis (20:18)	23	1B to 2A
13	No findings (20:44)	Pneumatosis, absent peristalsis (01:02, after midnight)	258	None to 2A

Study times represent the same day per patient unless otherwise indicated (crossing midnight). Patient cases 7 and 8 underwent abdominal ultrasounds prior to the initial radiograph

**Fig. 3:**
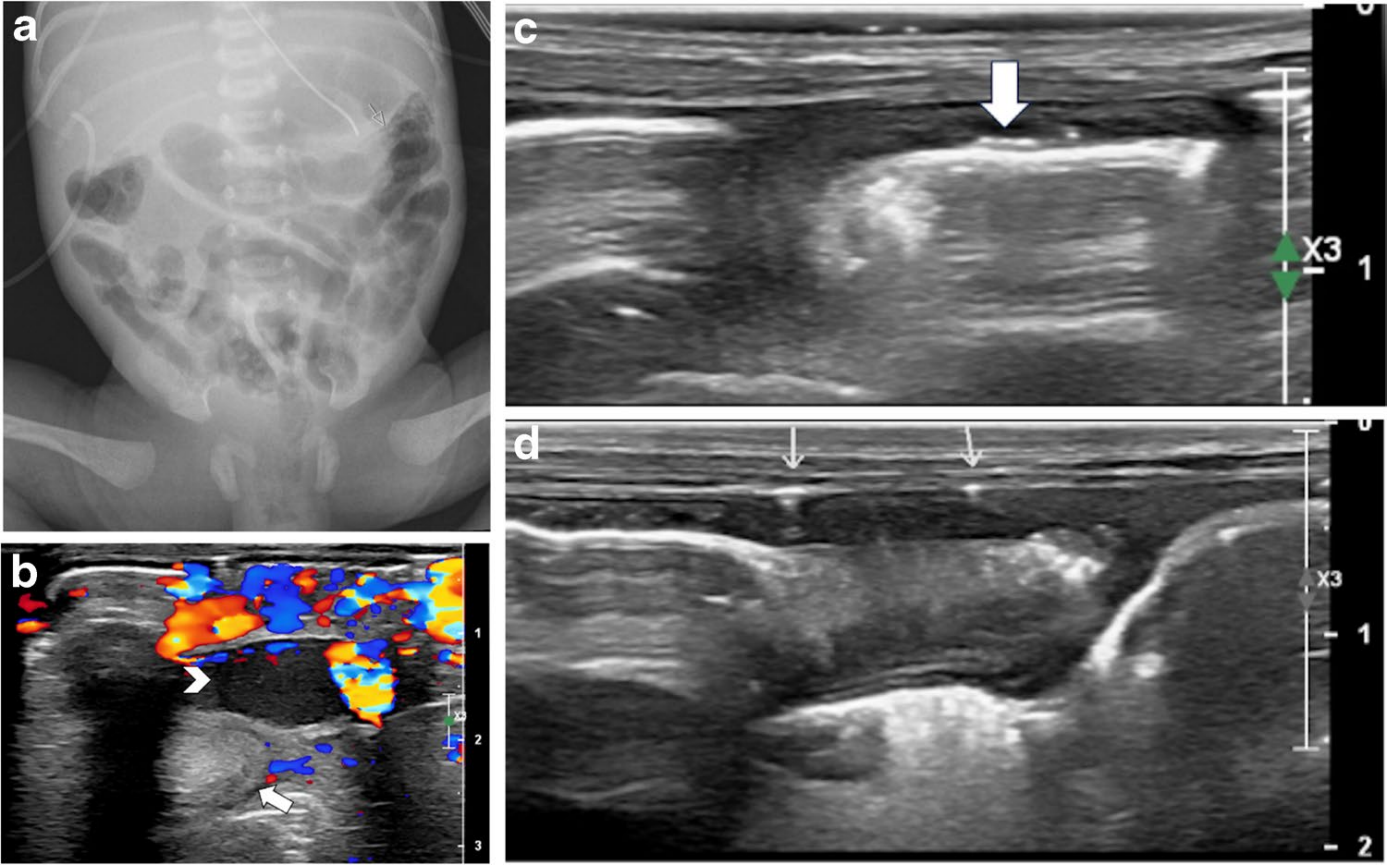
A 7-day-old preterm female born at 31 3/7 weeks (case 1 in Table 4). Abdominal ultrasound upgraded the modified Bell’s stage from 2 A to 3B. **a** An AP supine abdominal radiograph reveals pneumatosis in the right and left (*arrow*) upper quadrants. **b** Transverse abdominal ultrasound image of the right lower quadrant obtained 178 min later reveals bowel hypervascularity, bowel wall thickening (*arrow*), and complex interloop fluid (*arrowhead*). **c** Transverse ultrasound image of the right upper quadrant demonstrates gas within the bowel wall (*arrow*) with intraluminal gas deep to it. **d** Transverse ultrasound image of the right upper quadrant demonstrates small volume pneumoperitoneum (*arrows*) within complex free fluid

**Fig. 4:**
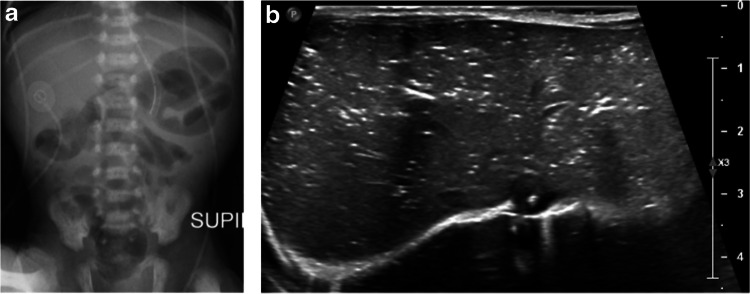
A 5-day-old male born at 32 0/7 weeks (case 2 in Table 4). Ultrasound imaging upgraded modified Bell’s stage from none to stage 2B. **a** An AP supine (SUPI) abdominal radiograph suggests mild separation of bowel loops but no distension, obstruction, or definitive pneumatosis. **b** Transverse ultrasound image of the right upper quadrant performed 31 min later reveals extensive portal venous gas throughout the liver

**Fig. 5:**
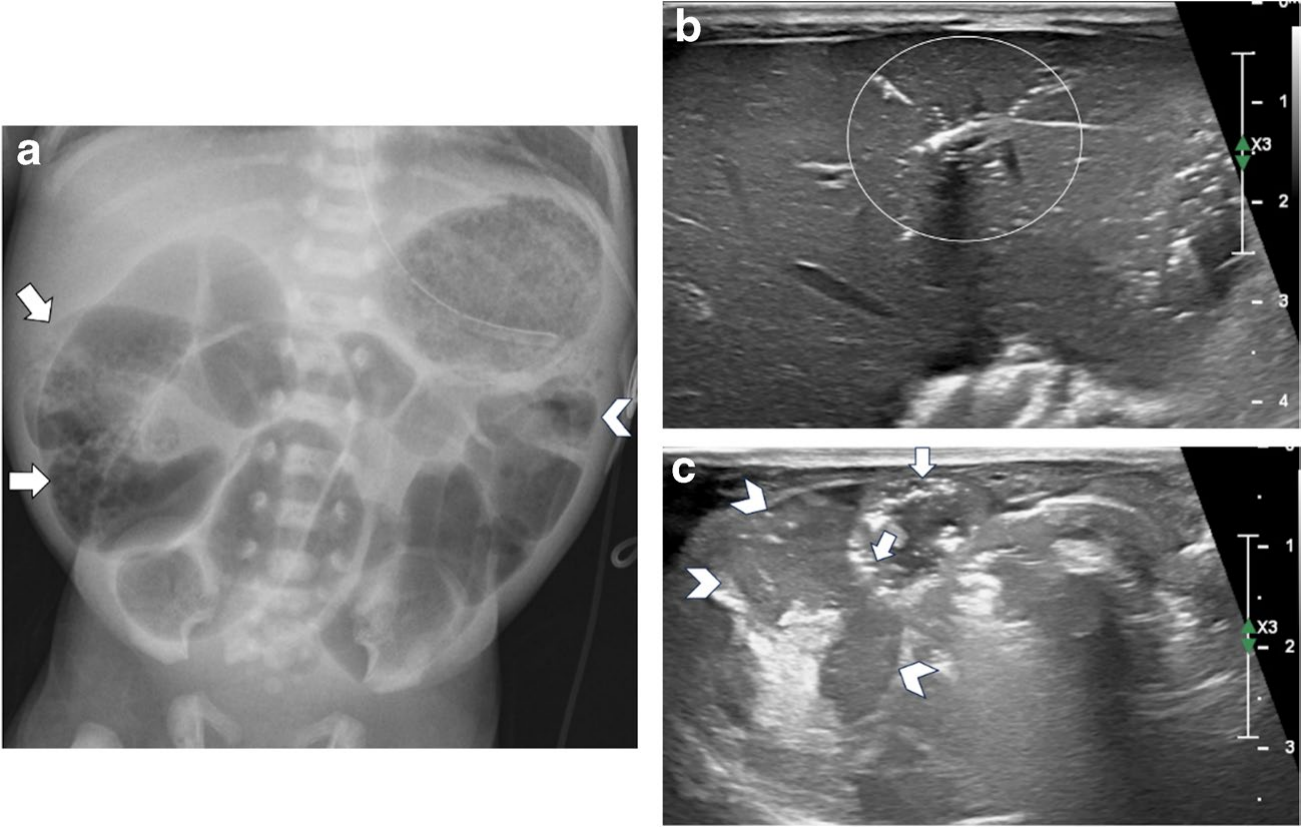
A 9-day-old female born at 29 4/7 weeks (case 10 from Table 4). Ultrasound imaging upgraded the modified Bell’s stage from 2 A to 3B. **a** An AP supine abdominal radiograph demonstrates gaseous distended bowel loops with pneumatosis on the right (*arrows*) and to a lesser degree, on the left (*arrowhead*). **b** Transverse midline abdominal ultrasound image performed 85 min later reveals a small amount of portal venous gas in the left hepatic lobe (*circle*). **c** Transverse right lower quadrant ultrasound image demonstrates pneumatosis (*arrows*) and complex, predominantly echogenic extraluminal fluid (*arrowheads*)

There were two (9%) false-negative ultrasounds when compared with abdominal radiography, with the initial abdominal radiographs showing pneumatosis. However, one false-negative abdominal ultrasound was performed 6 h after the initial radiograph. No infants with an outcome of surgery or death had false-negative ultrasounds. Conversely, initial radiography was negative for one infant who was subsequently diagnosed with pneumoperitoneum on ultrasound and one infant that was diagnosed with bowel perforation based on the volume of complex free fluid on abdominal ultrasound (Table 4). Figures 3, 4, and 5 provide radiographic and ultrasound images demonstrating imaging findings for patients 1, 2, and 10, respectively, previously referenced in Table 4.

## Discussion

This study is the first to evaluate a standardized protocol for obtaining and reporting abdominal ultrasound images following the implementation of a clinical guideline for universal adjunct abdominal ultrasound screening during the initial diagnostic evaluation for NEC. Before standardization, the feasibility and benefit of using abdominal ultrasound as an adjunct to abdominal radiography for diagnosing NEC were uncertain. Following implementation, adherence to the protocols was high in patients diagnosed with NEC, and no significant adverse events were associated with sonography for the diagnosis of NEC.

While prior studies have evaluated the benefits of adjunct abdominal ultrasound, a lack of standardization for its universal use has made it less apparent in which specific clinical scenarios the ultrasounds were obtained. The average length of time for performing the ultrasounds following protocol implementation was relatively brief: 13 min, and 23% of the ultrasounds were conducted between the hours of 19:00 and 07:00. These findings suggest that this protocol is feasible for adjunct abdominal ultrasounds to be obtained consistently during initial diagnostic evaluations, regardless of the time at which they occur.

Our findings are consistent with previous studies which postulated that ultrasonography is more sensitive than radiography in detecting pneumatosis intestinalis, portal venous gas, and possibly pneumoperitoneum [2, 5, 15–17]. A recent study by May et al. demonstrated the sensitivity and specificity of abbreviated abdominal ultrasonography for identifying high-risk findings of NEC, described as pneumoperitoneum, complex free fluid, and fluid collections, at 100% and 95%, respectively [2]. Although our study was unable to calculate the specificity of adjunct abdominal ultrasonography as it did not include negative cases of NEC, the sensitivity was high at 0.91. This was more sensitive than the initial abdominal radiograph for NEC (0.68). This is reassuring in the context of routine use, given that a 2018 meta-analysis reported sensitivities ranging from 0.27 to 0.48 for classic signs of NEC [18]. We speculate that universal practice with the abdominal ultrasound evaluation for NEC, especially to “rule in” highly suspected cases, resulted in improved sensitivity. This study highlights a significant portion of NEC cases (32%) that demonstrate positive ultrasound findings but no findings on initial abdominal radiographs. Additionally, another 23% of NEC cases had only indeterminate findings of possible pneumatosis as the sole finding on radiographs. In addition, 59% of cases resulted in a higher modified Bell’s stage from abdominal ultrasound findings when compared to the initial abdominal radiograph findings. These instances suggest that ultrasound is more sensitive than abdominal radiography alone in staging NEC. Both infants with pneumoperitoneum and bowel perforation by complex fluid collection on abdominal ultrasounds had negative radiographs for these findings. However, no cases of NEC in the abdominal ultrasound cohort had free air on radiography, so further studies are needed to investigate the sensitivity of universal adjunct abdominal ultrasonography for this finding.

In both cases, with false-negative initial abdominal radiographs and positive findings on abdominal ultrasound, universal screening with adjunctive abdominal ultrasound may have aided the earlier or more accurate staging of NEC. Precise diagnosis and staging of NEC may influence the choice of antibiotic therapy, duration of antibiotic treatment, or duration of bowel rest, depending on the center’s guidelines.

It is important to emphasize that, within this study, most of the ultrasound findings in NEC were not singularly present (e.g., only pneumatosis) but were present as a constellation of findings that informed the overall diagnosis and stage of NEC (e.g., pneumatosis, portal venous gas, and bowel wall thickening). Most clinicians agree that an isolated finding of pneumatosis in the absence of other signs and symptoms is insufficient to diagnose NEC. Pneumatosis can be present in non-NEC conditions (e.g., volvulus or benign pneumatosis coli) or it can be transient. While the presence of pneumatosis intestinalis can aid in staging NEC, it does not definitively correlate with disease severity. Therefore, multiple findings are crucial for achieving a high level of confidence in a diagnosis of NEC. Ultrasound was able to confirm the primary abdominal radiograph finding, but it often revealed multiple other findings consistent with NEC, thereby increasing provider confidence. A recent study by Le Cacheux et al. described an increased association with abdominal ultrasound findings, including thickening of the mesentery, increased echogenicity of intraluminal intestinal content, abnormalities of the abdominal wall, and poor definition of the intestinal wall, in infants with surgical NEC compared to those with medical NEC [20]. It may be valuable to incorporate these additional findings into adjunctive abdominal ultrasound protocols as part of a future feasibility study.

These results suggest that adjunctive abdominal ultrasound can aid in detecting findings during the initial evaluation of NEC and can be routinely applied in a relatively large academic neonatal care unit. Standardizing adjunct abdominal ultrasound as part of a multidisciplinary team may improve the reporting of findings in NEC compared to initial abdominal radiography, potentially leading to more accurate modified Bell’s staging of NEC or prognostication of medical versus surgical outcomes on initial evaluation.

We note that the pediatric radiology expertise at our center significantly aided in the design and implementation of the adjunct abdominal ultrasonography protocol for evaluating NEC. The Pediatric Radiology department at our center consists of six attending physicians who consistently use the created dictation template to report findings from adjunct ultrasounds, following the implementation of the protocol.

This study has several limitations, mainly due to its small sample size and retrospective design. The assessment of ultrasound sensitivity and specificity is limited because there is no definitive gold standard diagnostic test for NEC. Nevertheless, we can make relative comparisons between modalities. Diagnosis and staging of NEC can be subjective, but this is partly mitigated by a multidisciplinary team consensus on accurate staging using modified Bell’s criteria, determined by an independent panel of reviewers. Given the promising feasibility of establishing a guideline for universal adjunct abdominal ultrasonography for NEC at our single center, a multicenter trial would be helpful to further validate these findings. Typically, the number of NEC diagnoses made by ultrasound was relatively high and may reflect verification bias, as providers used ultrasound findings to guide their clinical diagnosis. We cannot calculate the specificity of abdominal ultrasound because of our unit guidelines. Since our center does not currently use abdominal ultrasound to “rule out” NEC, such as in cases of low suspicion, but mainly to confirm and stage cases with high suspicion where clinicians are already committed to an evaluation, therefore negative ultrasound results for NEC are unavailable. A separate study would be valuable for formally assessing this modality’s ability to rule out NEC in truly negative cases and for measuring changes in antibiotic use or NPO days, which comprise NEC treatment. Nonetheless, the rate of NEC at our center remains comparable to other academic centers nationwide and did not change during the study period. A larger sample size, especially from a population with a significantly lower NEC rate, might result in a higher false-positive rate and a lower positive predictive value. An extended period of follow-up after guideline implementation could provide additional insights.

## Conclusion

Standardizing universal, adjunct abdominal ultrasonography for the initial evaluation of NEC is a practical and promising approach that can be implemented using a multidisciplinary team, especially in the context of high clinical suspicion. Our findings contribute to the existing evidence that adjunctive abdominal ultrasonography is a valuable tool for diagnosing NEC compared to abdominal radiography alone. Multicenter studies incorporating adjunct abdominal ultrasound protocols to evaluate NEC may be beneficial in further validating our findings, which suggest a potential positive impact of universal adjunct abdominal ultrasound in improving the diagnosis of this devastating disease.

## Supplementary Information

Below is the link to the electronic supplementary material.

ESM 1 PDF (269 KB)

ESM 2 PDF (23.0 KB)

## Data Availability

No datasets were generated or analysed during the current study.
