# Supplementary material for: A standardized universal protocol for using adjunct abdominal ultrasound at the time of diagnosis for suspected necrotizing enterocolitis
Source: Pediatr Radiol. 2025 Oct 10;55(13):2823–31. doi: 10.1007/s00247-025-06408-x (PMC12708678; doi:10.1007/s00247-025-06408-x)
Supplement: Supplementary file 1 — PDF (269 KB) [file 247_2025_6408_MOESM1_ESM.pdf]

Medical University of South Carolina Neonatal Nursery Clinical Guideline: Management of Necrotizing Enterocolitis
Developed: January 2022
Latest revision: Vs.4: February 2025

Author Disclaimer: These are guidelines only and may not be applicable to populations outside of the MUSC Affiliated Hospitals. These guidelines do not represent official policy of Shawn Jenkins Children's Hospital, or the MUSC Department of Pediatrics, nor are they intended as practice guidelines or standards of care. Specific circumstances often dictate deviations from these guidelines. All users of this material should be aware of the possibility of changes to this guideline, which are routinely revised. Each practitioner assumes responsibility for their own decisions of care, and should not rely solely on the material published in this document.

Background [4, 5]

Necrotizing enterocolitis (NEC) is the most common gastrointestinal emergency and the leading surgical cause of death in the neonatal intensive care unit. NEC is primarily associated with prematurity. Immaturity of gastrointestinal tract function and immune-regulation mediates the risk of NEC. The average incidence of medical NEC in the United States is 5-7%.[1] In the extremely low birth weight (ELBW) < 1000g birth weight population, the incidence may be as high as 10%, and this group accounts for a higher proportion of severe surgical NEC.[1, 2] The incidence decreases as birth weight and gestational age increase.[2, 3] Most affected infants have received some form of enteral nutrition.[4] Overall, NEC is a culmination of inflammatory mucosal injury that results from multiple small insults specific to an early developmental period in the gastrointestinal tract.

Strategies to prevent NEC include:

- Early human milk feeding with standardized advancement[5-7]
- Minimizing unnecessary antibiotic exposure[8, 9]
- Minimizing acid blockade[10, 11]
- Removing central venous access as soon as possible[12, 13]
- Judicious use of blood products to reduce iatrogenic blood loss[14, 15]

The overall mortality rate for neonates with NEC is 25-30%. The risk of mortality is directly related to birth weight, gestational age, and need for surgery. For those treated non-operatively, the mortality varies directly with birth weight and gestational age, ranging from approximately 15% for 1250-1500g to > 40% for < 750g.[2] Short bowel syndrome and neurodevelopmental abnormalities are long-term sequalae of severe and surgical NEC.

Risk Factors

- Prematurity[16]
- ELBW/ VLBW/ IUGR[3]
- Twins/multiples[17]
- RDS, apnea, asphyxia, pulmonary hypertension[3]
- Enteral diet of low or no mother's own milk[18, 19]
- Congenital anomalies[3]
- Congenital heart disease,[20a] PDA[21, 22]

[a] Truncus arteriosus [OR 3.8], HLHS/aortopulmonary window [OR 6.8] and PGE ≥ 0.05 µg/kg/min [OR 3.9] are highest risk

- Hypothermia[23, 24b]
- Gastroschisis,[25] Hirschsprung's disease,[26, 27c] omphalocele[28]
- Medication:
    - acid suppression[11]
    - previous prolonged antibiotic exposures[8]
- Peripartum events: absent or reversed end diastolic umbilical arterial blood flow,[29] maternal eclampsia, fetal distress, and premature rupture of membranes[30]
- GI bacterial overgrowth/microbial dysbiosis[31]

Diagnosis

NEC has a broad spectrum of presentation and severity, and is defined by systemic signs and symptoms, abdominal exam, and radiographic findings.

- **Early signs of NEC:** apnea, bradycardia or unexplained new tachycardia, temperature instability, lethargy, feeding intolerance (defined as recurrent emesis, abdominal distension and/or decreased/absent bowel sounds), hypoglycemia/hyperglycemia, hypotension, worsening respiratory failure, bilious emesis, bloody stool, or an ileus pattern on x-ray.
- **Disease progression:** abdominal tenderness, absent bowel sounds, and radiographic signs (diffuse bowel dilation, pneumatosis intestinalis, portal venous gas, pneumoperitoneum, or a persistent fixed and dilated loop on serial images). *See imaging examples in the appendix.*
- **Advanced disease:** metabolic acidosis**,** thrombocytopenia, neutropenia, and disseminated intravascular coagulation.

Modified Bell Staging[d]

STAGE	Systemic Signs	Abdominal Signs	Radiologic Signs
**1. Suspected (Not confirmed)**	**A.** Temperature instability, apnea, bradycardia	Increased emesis, mild abdominal distention, occult blood in stool	Normal or mild ileus
	**B.** *Same as 1A*	Same as 1A, plus gross blood in stool	*Same as 1A*
**2. Definite: A. Mildly ill**	**A.** *Same as 1A*	Same as 1, plus absent bowel sounds, abdominal tenderness	Ileus, pneumatosis
**B. Moderately ill**	**B.** Same as 1, plus mild metabolic acidosis, mild thrombocytopenia	Same as 1, plus absent bowel sounds, definite abdominal tenderness, abdominal cellulitis, right lower quadrant mass	Same as 2A, plus portal vein gas, with or without ascites

---

[b] The risk of NEC is associated with VLBW infants with environmental hypothermia (low admission temperature), and less associated with therapeutic hypothermia in term infants, for whom the NEC rate is <1% (trials are currently on-going).

[c] Consider Hirschsprung disease in term infants with NEC

Charting: Following a diagnosis of NEC, a modified Bell's Stage should be assigned. Add NEC with staging as a diagnosis with appropriate ICD-10 code in the problem list in EPIC. Please note that "rule out NEC" should qualify as NEC, Stage I. In progress notes, please specify Stages.

**3. Advanced, Severely Ill**<br>**A. Bowel intact**	**A.** Same as 2B, plus hypotension, bradycardia, respiratory acidosis, metabolic acidosis, disseminated intravascular coagulation, neutropenia	Same as 1 and 2, plus signs of generalized peritonitis, marked tenderness, and distention of abdomen	Same as 2B, plus definite ascites
**B: Bowel perforated**	***B.*** *Same as 3A*	*Same as 3A*	*Same as 2B,* plus pneumoperitoneum

NEC Management[4, 32, 33]

- Consider intubation for patients on non-invasive positive pressure (nCPAP, niPPV) with abdominal distension and respiratory distress.[e]
- NPO[f] with Replogle[g] to low continuous suction for bowel decompression.[h]
- TPN with IL to maximize nutrition.[i] Consider adding Pepcid for stage 2B, which may be associated with a prolonged NPO period > 3 days.
- Close monitoring of vital signs, abdominal circumference, and urine output.
- Consider arterial access due to the need for frequent lab draws and for close BP surveillance.

Labs	All Suspected Patients	Patients ≥ 2B+ and Special Cases
Initial	Blood culture, urine culture[j], +CSF[k]<br>CBC, CRP, BMP	• ABG/VBG + lactate<br>• PT/INR/fibrinogen<br>• Intraoperative cultures (if surgical)
Q6	*Only previously abnormal labs[l]*	• CBC/BMP, space to q 12 then q 24 if normal

- GOAL is from order-time to receipt of antibiotics: < 1 hour
- For suspected NEC (Grade ≥ 2A)
    - Perform bedside huddle (MD/NNP/PA and bedside nurse) at time of recognition to streamline tasks, procedures and medication administration
    - Alert pharmacist on call if not already involved
    - MD/NNP/PA should be alerted by bedside nurse if cultures or antibiotics are delayed > 1 hour[m] after bedside huddle

---

[e] Increasing abdominal distension can result in the loss of lung volume and increase the need for positive-pressure ventilation during acute phase

[f] Refer to Post-NEC Refeeding Management below

[g] Use weight-based nursing guidelines for size (8F/10F)

[h] Current Argyle™ Replogle Suction Catheters do not need intermittent suction because of an extra side-port

[i] Hyperkalemia and oliguria/anuria is common in NEC; potassium should be decreased/removed from IV fluids

[j] Avoid suprapubic tap with acute abdominal exam

[k] Specifically, with neurological signs concerning for meningitis such as seizures, increased apnea or bradycardic events, hypotonia, lethargy, [unless thrombocytopenic (<100)]

[l] Optional to follow CRP q 24 hours

[m] Consider altering order of cultures for time-sensitivity of antibiotic receipt

[n] US has high specificity, but sometimes low sensitivity for NEC. Thus, it cannot "rule-out" NEC.

- Treat blood dyscrasias and electrolyte abnormalities per unit guidelines
- Films: Babygram and left lateral decubitus (for concern for pneumoperitoneum) q6h. Consider spacing interval once stable for 24 hours.
- Abdominal Ultrasound: Order limited AUS once, with indication: *evaluate for NEC*
    - Significant findings: pneumatosis, portal venous gas, bowel wall thickening/thinning, pneumoperitoneum, hyperemia, absent peristalsis and focal/complex fluid collection.
    - **Note:** Do not delay a NEC diagnosis by waiting for ultrasound results to confirm suspicious xray findings. If pneumatosis/portal venous gas/or pneumoperitoneum is possible on KUB, then treatment should be ordered.[n] Repeat abdominal US as clinically indicated.[o]

[o] No evidence exists to guide the management of repeat abdominal US in NEC

Medications	Choice	Length of treatment
Grade 1A-1B (unconfirmed)	PER LOS guideline	48 hours or per appropriate plan if cultures identify source
Grade 2A (no central line[n])	Zosyn IV	7 days[o]
Grade 2B (no central line[n])	Zosyn IV	10 days[o,p]
Grade 2 **+ central line**	Vancomycin IV and Zosyn IV	*Follow Grade 2A and 2B above*[q]
All Grade 3, Severe Surgical ± perforation	Vancomycin IV and Zosyn IV	14 days or per surgical team

- Consider the addition of fungal treatment if at high risk for fungal disease (e.g. history of fungal infection)

Consults:

- Pediatric Surgery
- Intestinal Rehab Team if:
  - Surgical NEC with more than 10% resection
  - An ostomy is placed
- Palliative Care Team if:
  Severe cases with major resections and/or NEC totalis

Indications for Surgical Intervention in NEC [1]

On-going collaboration is advised during decision-making regarding surgical intervention. These are potential indications:

- Pneumoperitoneum (most sensitive film is a lateral decubitus x-ray, but 1/3 of patients with perforation will not have free air evident on radiographs)
- Clinical deterioration (thrombocytopenia,[r] acidosis, severe hemodynamic instability)
- Abdominal compartment syndrome: abdominal distention with respiratory deterioration, ascites, edema, compromised lower extremity/global perfusion
- Abdominal wall meconium staining, cellulitis and/or edema (including scrotal edema in males)
- Abdominal mass on exam
- "Fixed loop"- persistent isolated dilated loop on serial XR

Pre-op preparation:

- Send a type and cross if not already obtained
- Check CBC, PT/PTT, fibrinogen and electrolytes if not recently done
- Transfuse blood products as needed (platelets if < 50k)

[n] Due to the risk of CONS bacteremia, use vancomycin in place of ampicillin if a central line is currently present (even if in place < 10 days)

[o] Unless persistent pneumatosis or progression of disease, then consider changing antibiotic regimen in consultation with pharmacist

[p] Up to 14 days at provider discretion due to severity of disease

[q] With ability to narrow to Zosyn if cultures are negative 72 hours, and clinically improving

[r] Thrombocytopenia is present in 30-50% of infants with NEC and in part (~50%) is caused by platelet binding by endotoxin produced by bacteria in blood or G.I. tract. A low or rapid fall in platelet count tends to be associated with sicker infants and a poorer prognosis, similar to those with more significant neutropenia. *A rapid fall of platelets from 150 to <100 is in 24 hours is associated with a 92% positive predictive value for intestinal gangrene* 37.

Ververidis M, Kiely EM, Spitz L, et al. The clinical significance of thrombocytopenia in neonates with necrotizing enterocolitis. Journal of Pediatric Surgery. 2001;36(5):799-803.

Laparotomy versus drain:

The preoperative diagnosis of NEC or spontaneous intestinal perforation modifies the impact of initial treatment (peritoneal drainage versus laparotomy) in ELBW patients.[38] Thus, the surgical plan should be developed via discussion between Pediatric Surgery and Neonatology teams. The location of surgical intervention (bedside/procedure room/operating room) will be on an individual basis and through a multidisciplinary discussion between the surgical, neonatology, and anesthesia teams.

NEC Totalis:

All NEC cases should be treated as potentially aggressive. NEC totalis describes operative findings in which at least 80% of the entire bowel is gangrenous. It is associated with nearly 100% mortality in infants < 1000g and nearly all survivors will have severe short bowel syndrome. Most surgeons suggest that aggressive care should be avoided, and if NEC totalis is discovered at operation a multi-disciplinary discussion with the neonatology and surgical teams should guide families toward goals of care. The palliative care team should be consulted if diagnosis is strongly suspected or confirmed.

Potential Complication post-NEC:

Intestinal strictures may result after NEC, particularly in patients who are managed medically. This is a result of fibrotic healing and scarring in an area of ischemia. Strictures can occur anywhere from 3-6 weeks after the onset of NEC and require a high index of suspicion. Should feeding intolerance be encountered, discussion with the surgical team regarding the timing of lower and then upper contrast studies to diagnose a stricture should occur. Because the highest incidence of stricture formation is in the colon (80%) and ileum (15%), a contrast enema is the initial study of choice. An early-onset stricture (before 6 weeks) may resolve spontaneously; others generally require surgical resection.

Management of Refeeding Neonates after NEC
(Per NEC Refeeding Guideline)

Background:

For infants with necrotizing enterocolitis (NEC) treated non-operatively, no consensus exists on the optimal fasting period prior to reintroducing feeds after NEC. Two previously published studies and our own data from our NEC patients from the past 10 years show that earlier refeeding does not increase the rate of NEC recurrence, stricture or death and may have benefits including shorter duration of TPN, fewer PICC line days and CLABSIs, and shorter hospital LOS. One study had a median of 4 days vs. 10 days, a second study compared 5 days; our study examined 7 days. We performed a survey of our Neo faculty, fellows and NNPs, Ped Surgery faculty and NPs to arrive at a proposal as to when to start feeds after NEC. After implementing the agreed upon algorithm, we published our findings that following the consensus-based earlier refeeding guidelines decreased time to full feeds and central line days without significant increase in adverse events.

Post-NEC Refeeding Management[39, 40]

Introduction of post-NEC feeding will be based on the following paradigm[41] (Table 1 below):

<table>
<tr><td>NEC Stage</td><td>Restart feeds* on day below assuming:
1. Infant is clinically systemically well
2. Infant has normal abdominal exam 12 hours after Replogle has been replaced by an OG. It is recommended that the Replogle is placed to gravity 24 hours prior to restarting feeds and changed to OG to vent 12 hours prior to restarting feeds.</td></tr>
<tr><td>Stage 1**</td><td>At the discretion of the clinical team</td></tr>
<tr><td>Stage IIA</td><td>3 days after NEC diagnosed</td></tr>
<tr><td>Stage IIB</td><td>3 days from last pneumatosis</td></tr>
<tr><td>Stage IIIA</td><td>7 days from last pneumatosis***</td></tr>
<tr><td colspan="2">*Feeds will be mother’s own or donor milk (regardless of weight/GA) using the post-NEC feeding protocols in Epic with the caveat that the protocol can be advanced if clinically appropriate (e.g. Stage I babies).

• For weight </= 1000g (15 days): trophic feeds of 12ml/kg x 5 days, advance by 17.5ml/kg, fortify at 99.5ml/kg (in two steps).
• For weight > 1000g (10 days): trophic feeds of 12ml/kg x 3 days, advance by 25ml/kg, fortify at 112ml/kg (in two steps).

**Do not need to document A vs. B for this stage.
***With the option to start trophic feeds as soon as 72h after last pneumatosis if the clinical team chooses.</td></tr>
</table>

References

1. Han SM, Hong CR, Knell J, et al. Trends in incidence and outcomes of necrotizing enterocolitis over the last 12 years: A multicenter cohort analysis. Journal of Pediatric Surgery. 2020;55(6):998-1001.
2. Walsh MC, Bell EF, Kandefer S, et al. Neonatal outcomes of moderately preterm infants compared to extremely preterm infants. Pediatric Research. 2017;82(2):297-304.
3. Yee WH, Soraisham AS, Shah VS, et al. Incidence and Timing of Presentation of Necrotizing Enterocolitis in Preterm Infants. Pediatrics. 2012;129(2):e298-e304.
4. Association APS. Not a Textbook, Up To Date Pediatric Surgery Curriculum Available: https://www.pedsurglibrary.com/apsa/index/Pediatric-Surgery-NaT/Disorders
5. Jasani B, Patole S. Standardized feeding regimen for reducing necrotizing enterocolitis in preterm infants: an updated systematic review. J Perinatol. 2017;37(7):827-833.
6. Committee AB, Eidelman AI, Schanler RJ, et al. Breastfeeding and the Use of Human Milk. Pediatrics. 2012;129(3):e827-e841.
7. Brotschi B, Baenziger O, Frey B, et al. Early enteral feeding in conservatively managed stage II necrotizing enterocolitis is associated with a reduced risk of catheter-related sepsis. J Perinat Med. 2009;37(6):701-705.
8. Kuppala VS, Meinzen-Derr J, Morrow AL, et al. Prolonged Initial Empirical Antibiotic Treatment is Associated with Adverse Outcomes in Premature Infants. The Journal of Pediatrics. 2011;159(5):720-725.
9. Alexander VN, Northrup V, Bizzarro MJ. Antibiotic Exposure in the Newborn Intensive Care Unit and the Risk of Necrotizing Enterocolitis. The Journal of Pediatrics. 2011;159(3):392-397.
10. Romaine A, Ye D, Ao Z, et al. Safety of histamine-2 receptor blockers in hospitalized VLBW infants. Early Hum Dev. 2016;99:27-30.
11. Terrin G, Passariello A, De Curtis M, et al. Ranitidine is associated with infections, necrotizing enterocolitis, and fatal outcome in newborns. Pediatrics. 2012;129(1):e40-45.
12. Rozé JC, Ancel PY, Lepage P, et al. Nutritional strategies and gut microbiota composition as risk factors for necrotizing enterocolitis in very-preterm infants. The American journal of clinical nutrition. 2017;106(3):821-830.
13. Berkhout DJ, Klaassen P, Niemarkt HJ, et al. Risk factors for necrotizing enterocolitis: a prospective multicenter case-control study. Neonatology. 2018;114:277-284.
14. MohanKumar K, Namachivayam K, Song T, et al. A murine neonatal model of necrotizing enterocolitis caused by anemia and red blood cell transfusions. Nature communications. 2019;10(1):1-17.
15. Kirpalani H, Bell EF, Hintz SR, et al. Higher or lower hemoglobin transfusion thresholds for preterm infants. New England Journal of Medicine. 2020;383(27):2639-2651.
16. Neu J. Necrotizing enterocolitis: the search for a unifying pathogenic theory leading to prevention. Pediatric Clinics. 1996;43(2):409-432.
17. Burjonrappa SC, Shea B, Goorah D. NEC in Twin Pregnancies: Incidence and Outcomes. J Neonatal Surg. 2014;3(4):45.
18. Abrams SA, Schanler RJ, Lee ML, et al. Greater mortality and morbidity in extremely preterm infants fed a diet containing cow milk protein products. Breastfeed Med. 2014;9(6):281-285.
19. Chetta KE, Schulz EV, Wagner CL. Outcomes improved with human milk intake in preterm and full-term infants. Semin Perinatol. 2021;45(2):151384.
20. McElhinney DB, Hedrick HL, Bush DM, et al. Necrotizing Enterocolitis in Neonates With Congenital Heart Disease: Risk Factors and Outcomes. Pediatrics. 2000;106(5):1080-1087.
21. Shteinberg M, Boyd J, Aliberti S, et al. What is important for people with nontuberculous mycobacterial disease? An EMBARC-ELF patient survey. ERJ Open Res. 2021;7(1).

22. Dollberg S, Lusky A, Reichman B, et al. Patent ductus arteriosus, indomethacin and necrotizing enterocolitis in very low birth weight infants: a population-based study. Journal of pediatric gastroenterology and nutrition. 2005;40(2):184-188.
23. Tay VY, Bolisetty S, Bajuk B, et al. Admission temperature and hospital outcomes in extremely preterm infants. J Paediatr Child Health. 2019;55(2):216-223.
24. Gale C, Longford NT, Jeyakumaran D, et al. Feeding during neonatal therapeutic hypothermia, assessed using routinely collected National Neonatal Research Database data: a retrospective, UK population-based cohort study. The Lancet Child & Adolescent Health. 2021;5(6):408-416.
25. Snyder CL. Outcome analysis for gastroschisis. Journal of Pediatric Surgery. 1999;34(8):1253-1256.
26. Till H, Castellani C, Moissl-Eichinger C, et al. Disruptions of the intestinal microbiome in necrotizing enterocolitis, short bowel syndrome, and Hirschsprung's associated enterocolitis. Frontiers in Microbiology. 2015;6.
27. Raboei EH. Necrotizing enterocolitis in full-term neonates: is it aganglionosis? European journal of pediatric surgery. 2009;19(02):101-104.
28. Ravikanth R. Prenatal diagnosis of giant omphalocele on ultrasonography. Apollo Medicine. 2021;18(4):318-318.
29. Malcolm G, Ellwood D, Devonald K, et al. Absent or reversed end diastolic flow velocity in the umbilical artery and necrotising enterocolitis. Archives of disease in childhood. 1991;66(7 Spec No):805-807.
30. Hall NJ, Eaton S, Pierro A. Necrotizing enterocolitis: Prevention, treatment, and outcome. Journal of pediatric surgery. 2013;48(12):2359-2367.
31. Tarracchini C, Milani C, Longhi G, et al. Unraveling the Microbiome of Necrotizing Enterocolitis: Insights in Novel Microbial and Metabolomic Biomarkers. Microbiology Spectrum.9(2):e01176-01121.
32. Blackwood BP, Hunter CJ, Grabowski J. Variability in Antibiotic Regimens for Surgical Necrotizing Enterocolitis Highlights the Need for New Guidelines. Surg Infect (Larchmt). 2017;18(2):215-220.
33. Neonatal Infections. Springer International Publishing 2018.
34. Turcios-Ruiz RM, Axelrod P, John KS, et al. Outbreak of necrotizing enterocolitis caused by norovirus in a neonatal intensive care unit. The Journal of pediatrics. 2008;153(3):339-344.
35. Panico MG, D'Anna A, Pezzulo L, et al. A cluster of necrotizing enterocolitis in neonatal intensive care unit of one of the hospitals in Salerno, Italy. Senses and Sciences. 2014;1(4).
36. Tan YY, Quek BH, Thoon KC, et al. Successful containment of horizontal enterovirus infection in a neonatal unit in Singapore through diagnosis by polymerase chain reaction (PCR) and direct sequence analysis. Journal of Infection and Public Health. 2020;13(10):1556-1561.
37. Ververidis M, Kiely EM, Spitz L, et al. The clinical significance of thrombocytopenia in neonates with necrotizing enterocolitis. Journal of Pediatric Surgery. 2001;36(5):799-803.
38. Blakely ML, Tyson JE, Lally KP, et al. Initial Laparotomy Versus Peritoneal Drainage in Extremely Low Birthweight Infants With Surgical Necrotizing Enterocolitis or Isolated Intestinal Perforation: A Multicenter Randomized Clinical Trial. Annals of surgery. 2021;274(4):e370-e380.
39. Patel EU, Wilson DA, Brennan EA, et al. Earlier re-initiation of enteral feeding after necrotizing enterocolitis decreases recurrence or stricture: a systematic review and meta-analysis. J Perinatol. 2020;40(11):1679-1687.
40. Patel EU, Head WT, Rohrer A, et al. A quality improvement initiative to standardize time to initiation of enteral feeds after non-surgical necrotizing enterocolitis using a consensus-based guideline. J Perinatol. 2022.
41. Bohnhorst B, Muller S, Dordelmann M, et al. Early feeding after necrotizing enterocolitis in preterm infants. J Pediatr. 2003;143(4):484-487.
