# Supplementary material for: A standardized universal protocol for using adjunct abdominal ultrasound at the time of diagnosis for suspected necrotizing enterocolitis
Source: Pediatr Radiol. 2025 Oct 10;55(13):2823–31. doi: 10.1007/s00247-025-06408-x (PMC12708678; doi:10.1007/s00247-025-06408-x)
Supplement: Supplementary file 2 — PDF (23.0 KB) [file 247_2025_6408_MOESM2_ESM.pdf]

Online Resource 2: Negative Result Standardized Pediatric Radiology Dictation Template for Limited Abdominal Ultrasound for the Diagnosis of Necrotizing Enterocolitis.

EXAMINATION:

ACCESSION NUMBER:

INDICATION:

COMPARISON: None.

TECHNIQUE: Multiple sonographic images were obtained of the abdomen utilizing a multihertz transducer.

FINDINGS:

Bowel:
- Wall thickening: Absent.
- Dilation: Absent.
- Pneumatosis intestinalis: Absent.
- Perfusion: Normal.

Abdominal fluid:
- Free fluid: Absent.
- Fluid complexity: Simple.
- Focal fluid collection: Absent.

Portal venous gas: Absent.

Pneumoperitoneum: None demonstrated.

IMPRESSION:

No specific findings of necrotizing enterocolitis.
